# Damage Response Protein Buys Time for Bacterial DNA Repair

**DOI:** 10.1371/journal.pbio.0020325

**Published:** 2004-09-07

It is often said that after a nuclear catastrophe, cockroaches will inherit the earth, because they are so resistant to the harmful effects of ionizing radiation. But should the unthinkable come to pass, the bacterium Deinococcus radiodurans is likely to outlast even the cockroach. Its ability to endure radiation is truly impressive: it can withstand a dose a thousand times that which will kill a human. How it accomplishes this phenomenal feat of survival is the subject of a study in this issue by John Battista and colleagues at Louisiana State University in Baton Rouge and at the University of Wisconsin at Madison.[Fig pbio-0020325-g001]

**Figure pbio-0020325-g001:**
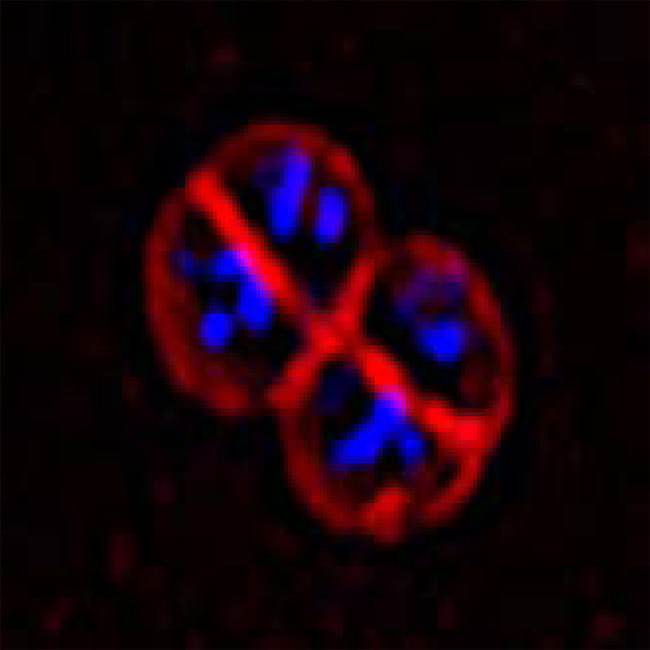
D. radiodurans R1

While radiation damages many cellular components, it is the fracturing of the cell's DNA that is the most harmful. DNA breaks can be repaired, but in doing so, the cell is racing against time. The exposed free ends of the DNA invite digestion by the cell's own enzymes, called exonucleases. If the DNA is not stitched back together quickly enough, the exonucleases will degrade it past the point of repair, and the cell will ultimately succumb. Large doses of radiation can fracture a chromosome in thousands of places, far in excess of the repair ability of most cells. D. radiodurans, however, largely prevents exonuclease digestion, an ability which has previously been shown to be linked to the activity of a gene with the rather uninformative name of DR0423. But how, exactly, does this gene accomplish this life-saving feat?

To answer this question, Battista and colleagues first showed that, following radiation exposure, DR0423 was upregulated 20- to 30-fold, and that deletion of the gene renders D. radiodurans susceptible to ionizing radiation. Together, these results clearly indicate that the DR0423 gene product is critical for protecting the bacterium. Based on this, they dubbed the gene *ddrA*, for “DNA damage response.” They also found that DdrA, the protein encoded by *ddrA*, binds to single-stranded fragments of DNA, exactly like those found at the broken ends of the DNA double helix when damaged by radiation. Finally, they showed that when DdrA bound to these broken ends, they were protected from digestion by exonucleases.

An important question about this system is what it is actually good for. Since the level of radiation tolerated by the bacterium is found nowhere on earth, of what use is such an efficient DNA protection system? The answer might be that it also protects D. radiodurans from the effects of desiccation, a condition much more common in the life of a bacterium, and one which also induces widespread DNA damage. While *ddrA* cannot prevent the damage, it can preserve the DNA from degradation until conditions once again allow the bacterium to function, and repair its DNA.

